# Effectiveness of an individualized home-based physical activity program in surgery-free non-endarterectomized asymptomatic stroke patients: a study protocol for the PACAPh interventional randomized trial

**DOI:** 10.1186/s13063-022-06061-x

**Published:** 2022-02-14

**Authors:** Mathilde Mura, Emeraude Rivoire, Leila Dehina-Khenniche, Michèle Weiss-Gayet, Bénédicte Chazaud, Camille Faes, Philippe Connes, Anne Long, Chantal L. Rytz, Pauline Mury, Lidia Delrieu, Etienne Gouraud, Marine Bordet, Nellie Della Schiava, Patrick Lermusiaux, Matthieu Arsicot, Antoine Millon, Vincent Pialoux

**Affiliations:** 1 Atherosclerosis, Thrombosis and Physical Activity, LIBM EA7424, Université Lyon 1, University of Lyon, Lyon, France; 2 Vascular Medicine Department, Hopital Edouard Herriot, Hospices Civils de Lyon, Lyon, France; 3 Vascular and Endovascular Surgery Department, Hopital Louis Pradel, Hospices Civils de Lyon, Lyon, France; 4 Stem Cell Environment and Skeletal Muscle Homeostasis, Institut NeuroMyoGene, CNRS UMR 5310, INSERM U1217, Université Claude Bernard Lyon 1, University of Lyon, Lyon, France; 5 Vascular Biology and Red Blood Cell, LIBM EA7424, Université Lyon 1, University of Lyon, Lyon, France; 6 Libin Cardiovascular Institute, Cumming School of Medicine, University of Calgary, 3230 Hospital Drive NW, Calgary, Alberta Canada; 7 Center for Research, Montreal Heart Institute, Montreal, Quebec, Canada; 8 Residual Tumor & Response to Treatment Laboratory, RT2Lab, Translational Research Department, INSERM, U932 Immunity and Cancer, Institut Curie, Paris University, Paris, France; 9 Electrical Engineering and Ferroelectrical Laboratory, INSA Lyon, Lyon, France

**Keywords:** Atherosclerosis, Physical activity, Intraplaque hemorrhage, Monocyte phenotype, Physical fitness, Randomized controlled trial

## Abstract

**Background:**

Carotid atherosclerotic plaques remain silent until their rupture, which may lead to detrimental ischemic events such as strokes. This is due, in part, to intraplaque hemorrhages (IPH) and the resulting inflammatory processes, which may promote carotid plaque vulnerability. Currently, the benefits of carotid endarterectomy remain unclear for asymptomatic patients. Interestingly, the completion of physical activity (PA) may have beneficial effects; however, the paucity of current data warrants robust longitudinal interventions. We therefore aim to study the effects of a 6-month longitudinal personalized home-based PA program on IPH, biological, and inflammatory markers in asymptomatic stroke patients.

**Methods:**

Eighty patients (≥ 18 years old) will be recruited for the Physical Activity and Carotid Atherosclerotic Plaque Hemorrhage (PACAPh) clinical trial from the *Hospices Civils de Lyon*. Patients will be eligible if they present with carotid stenosis ≥ 50% and are asymptomatic from any ischemic events for at least 6 months. Recruited patients will be randomized into either a PA or a control group, and assessed at baseline and after 6 months. At both time points, all patients will be assessed using magnetic resonance imaging to assess IPH, blood sampling to measure inflammatory markers and monocytic phenotyping, PA and sedentary behavior questionnaires, 6-min walking test, and maximal isometric quadricep contraction test. The randomized PA intervention will consist of reaching a daily walking step goal individually tailored to each patient. Steps will be collected using a wirelessly connected wristband. The number of steps completed by individuals in the PA group will be re-evaluated bimonthly to encourage walking habits.

**Discussion:**

The PACAPh study is the first of its kind representing a feasible, easily accessible therapeutic strategy for asymptomatic stroke patients. We hypothesize that the personalized home-based PA program will reduce IPH and modulate inflammatory and biological parameters in patients presenting with carotid plaques. If the results of the PACAPh study prove to be beneficial on such health parameters, the implementation of such kind of intervention in the daily treatment of these patients would be an advantageous and cost-effective practice to adopt globally.

**Trial registration:**

This study has been approved by the National Ethics Committee (IDRCB:2019-A01543-54/SI:19.06.21.40640). ClinicalTrials.gov NCT04053166

## Background

Stroke is one of the most significant causes of death worldwide and is the leading cause of long-term disability in Western countries [1]. More than 85% of strokes are considered ischemic (i.e., reduced blood flow in select brain regions), and at least 20% of ischemic strokes result from the rupture of an unstable plaque located at the carotid artery bifurcation [1]. In symptomatic stroke patients, the removal of the unstable carotid plaque via carotid endarterectomy (CEA) can reduce the risk of stroke recurrence [2, 3]. However, the benefits of CEA treatment in asymptomatic stroke patients remain unclear [4] as CEA procedures have shown no difference in ipsilateral stroke rates after 6 months [3] to 1 year [2] post-CEA compared to the sole preferred medical treatment. Consequently, European guidelines suggest that patients who do not present with clinical symptoms (i.e., ischemic events) or plaque vulnerability factors (i.e., intraplaque hemorrhage [IPH], lipid-rich necrotic core, or thinning of the fibrous cap determined by imaging) should not undergo CEA [5].

The accumulation of lipids at the bifurcation of large arteries may elicit an inflammatory response [6] leading to diapedesis and accumulation of circulating monocytes, thus forming a plaque. As such, monocytes can thereby differentiate into macrophages and engulf oxidized low-density lipoproteins, to form foam cells [7] and promote the expansion of atherosclerotic plaques. This pro-inflammatory, hypoxic, and pro-oxidant environment within a plaque can further increase its vulnerability. Additionally, the formation of leaky neovessels that spread from the adventitia can increase the risk of IPH [8–10], which can sustain the local pro-inflammatory environment by continuously attracting circulating monocytes [11]. IPH also increases the volume of the necrotic core, leading to fibrous cap thinning [12, 13] which may also be promoted by macrophages residing within the plaque. Thus, the plaque may disintegrate and dislodge, causing a leakage of plaque contents into circulation, and thereby promote a general pro-thrombotic state [14].

According to the current European Society for Vascular Surgery Guidelines [5], there is a significant need to maintain plaque stability in asymptomatic stroke patients with carotid stenosis > 50% and an unfavorable risk/benefit ratio of undergoing CEA. Although these patients are usually treated with statins, angiotensine-converting enzyme inhibitor, or anti-platelets [5], pharmacological therapeutics have not been proven to efficiently stabilize such plaques [15].

It has been demonstrated that aerobic physical activity (PA) may decrease both low-grade inflammation in cardiovascular patients [16] and the pro-oxidant state in atherosclerotic patients [17]. A recent cross-sectional study showed a lower prevalence of IPH [18] and a reduced red blood cell (RBC) aggregation [19] in patients with higher PA levels. Furthermore, we recently observed that low-intensity PA is associated with a reduced rate of pro-inflammatory and phagocytic monocytes (CD14^++^/CD16^−^; CD14^++^/CD16^+^), as well as an increased rate of healing monocytes in carotid atherosclerotic patients (CD14^+^/CD16^++^) [20]. The vascular benefits of maintaining a high level of PA in atherosclerotic patients have only been explored in cross-sectional [17–20] or interventional studies assessing non-carotid atherosclerotic patients [14, 21], thereby limiting their direct applicability to carotid atherosclerotic patients.

In this context, PA may be an appropriate and effective therapeutic approach for atherosclerotic patients with carotid plaques. Therefore, it is of great importance to investigate the specific longitudinal effects of PA in patients with vulnerable non-operable carotid plaques.

In comparison with PA programs performed in rehabilitation centers, home-based PA has shown superlative rates of adherence, both during interventional studies and after study cessation [22, 23]. Home-based PA interventions may address the social and territorial inequities seen with PA completed at other locations and institutions [24–30]. Further, home-based PA adherence is increased when exercise is completed using connected electronic devices [31] (e.g., Fitbit), supplemented with individualized PA goal setting. As such, with highly tailored and attainable PA goals that are adapted to physical capabilities, individuals are more likely to be motivated in reaching their PA goals on a regular basis. Further, a recent meta-analysis indicated that self-monitoring of PA habits improved steps per day counts in patients with cardiovascular diseases [32]. We have previously implemented this experimental design in metastatic breast cancer patients with a 94% participation rate and 98% compliance [33]. Further, we recently reported that walking accounted for almost all of the reported moderate-intensity PA practiced by carotid atherosclerotic patients [18]. Therefore, a personalized PA program coupled with routine self-monitoring may improve the PA habits and thereby the health outcomes in these patients.

The Physical Activity and Carotid Atherosclerotic Plaque Hemorrhage (PACAPh) clinical trial is the first individualized home-based PA randomized interventional study that primarily aims to assess the effects of a PA intervention on the levels of carotid IPH using magnetic resonance imaging (MRI) in non-endarterectomized asymptomatic stroke patients. The second aim is to investigate the effects of the PA intervention on (1) the monocytic phenotype; (2) hematology and blood rheology; (3) coagulation profiles; (4) plasma oxidative stress levels, anti-oxidant enzymes activity, and inflammation; (5) average two-week daily step counts; (6) fitness and limb strength; (7) PA and sedentary behavior levels; (8) nutrition and quality of life; and (9) the development/progression of comorbidities.

## Methods

### Study design

**Fig. 1:**
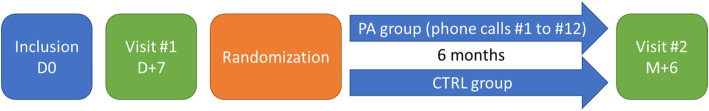
Flowchart of the PACAPh study

The PACAPh study is a longitudinal, interventional, monocentric, randomized controlled study sponsored by the *Hospices Civils de Lyon* (Lyon, France) (Fig. 1).

### Study population

All patients must meet the following criteria to be eligible: (1) male or female; (2) ≥ 18 years old; (3) presence of a carotid atherosclerotic plaque detected by echo doppler exam, with a stenosis ≥ 50% (North American Symptomatic Carotid Endarterectomy Trial [NASCET]); (4) without CEA surgery indication; (5) treated at the vascular surgery unit at the *Hospices Civils de Lyon*; (6) no contraindication to PA (World Health Organization Performance Status < 2); (7) available and motivated to partake in the study for a 6-month duration; (8) able to understand, read, and write in French; (9) valid health insurance affiliation; and (10) able to use a compatible smartphone or tablet (i.e., iOS or Android) and have access to an internet connection.

Exclusion criteria include (1) transient ischemic attack or stroke within the past 6 months; (2) previous carotid homolateral surgery or cervical irradiation; (3) cancer of any kind, heart failure, or human immunodeficiency virus seropositivity; (4) coronary diseases: silent ischemia, angora, or acute coronary syndrome; (5) uncontrolled neurological diseases: epilepsy, Parkinson, or Alzheimer’s disease; (6) renal failure (creatinine clearance according to Cockroft < 30 mL/min); (7) ProHance® 0.1 mmol/kg (Gadoteridol, Bracco Imaging S.P.A., Colleretto Giacosa, Italy) contraindications and precautions for use (i.e., hypersensitivity to the active principle or one of the ProHance® constituent, renal failure with clearance < 30 mL/min/1.73 m^3^, increased risk of convulsions during the examination for epileptic and/or brain damaged patients), or if patients are currently pregnant (> 40 UI human chorionic gonadotropin or breastfeeding); (8) MRI contraindications (ferromagnetic equipment: pacemaker, implantable devices, artificial heart valve, cochlear implant, neurostimulators, implanted automated injection equipment, metallic intraocular foreign body, vascular and/or neurosurgical clips); (9) homolateral carotid occlusion; (10) homolateral intracranial stenosis; (11) adult guardianship, curatorship, custody of the court, or protected by French law; (12) incapacity to explicitly express consent; (13) altered mental function and/or altered body function resulting in the incapacity to express personal will; and (14) unable to complete follow-up due to medical, social, geographical and/or psychological reasons for the duration of the study.

### Recruitment

Eighty patients will be recruited in the Louis Pradel cardiology hospital of the *Hospices Civils de Lyon* and will be followed for a period of 6 months. The study protocol and the patient’s rights for human clinical research will be proposed and explained to the patients by their vascular surgeon. Those patients willing and able to agree to the terms of study enrollment will be included after written informed consent is obtained. The investigator and the patient will sign two copies of the consent form, and the investigator and patient will each keep one copy. On the consent form, participants will be asked if they agree to the use of their data should they choose to withdraw from the trial at any time. Participants will also be asked for permission for the research team to share relevant data with other personnel from the universities taking part in the research study or from regulatory authorities, where relevant. This trial involves collecting biological specimens for storage.

### Randomization

At the recruitment visit, patients will be randomly assigned (1:1 ratio) using an electronic case report form (eCRF; ENNOV Clinical, CSOnline 7.5.720.1) to either (1) a 6-month individualized home-based PA intervention with a FitBit activity tracker or (2) a control group without an activity tracker or PA intervention (detailed below).

### Six-month physical activity intervention

#### Interventional group

Patients randomized into the PA intervention group will receive a “FitBit Alta HR” activity tracker (FitBit, CA, USA) to wear during daytime hours (~ 7 h/day) throughout the 6-month intervention period. The final PA goal of this study is for patients to meet an average of 6000 steps/day or—if they already meet 6000 steps/day at inclusion—to increase the average steps/day count by 30% as compared to their baseline average steps/day count. During the first visit, the clinical research assistant will download the “Fitbit” mobile application onto the patients’ respective mobile or tablet device and connect the tracker to laboratory equipment in order to transfer data via Bluetooth. The PA instructor will give patients instructions on how to use both the activity tracker and mobile application. Data on patients’ activity will be immediately available to the PA instructor through the “FitBit” dashboard. After visit #1, patients will wear the activity trackers without any PA guidance or objectives provided by study personnel. Patients will be contacted via telephone by the PA instructor every 14 days throughout the 6-month intervention. During the first phone call, the PA instructor will propose a personalized daily step goal according to the number of daily steps voluntarily done by the patient in the first 14 days that is both within the patients’ capability and preference level, as well as is agreed upon by both the PA instructor and patient. Then, during each of the successive phone calls (calls #2–12) and based on the daily step count achieved during the 14 preceding days, the PA instructor will adapt the future daily step count goals as appropriate to ensure the final daily step goal will be reached. The goal may be increased, maintained, or even decreased if the previous goal is too difficult to reach. During phone calls, the PA instructor will also inquire about any other potential problems experienced by the patient with respect to the tracker, the application, and/or the PA practice. Throughout the intervention, a phone line will be available for patients to contact the study team for any questions or any concerns.

#### Control group

Patients randomized into the control group will only wear the activity tracker for 14 days following visit #1 and 14 days before visit #2 in order to measure daily walking habits and step counts. No recommendations regarding PA will be proposed during the study period. They will continue to be followed according to their usual care.

### Evaluations modalities

Firstly, the investigator surgeon will complete a clinical and physical examination in order to deliver a PA non-contraindication certificate for each patient. Patients will then have two separate visits at the hospital dedicated to the study. A first baseline study visit (#1; Fig. 1) will be scheduled within a month of the granting of the PA non-contraindication certification in order to perform the para-clinical measurements. At the end of the study, all tests will be repeated during visit #2 and compared with those performed during visit #1. Upon completion of visit #2, enrolled patients will be terminated from the study. For both study visits (#1 and #2), the PA instructor will perform the physical assessments, and blood samples will be taken by a registered nurse. Intercurrent care and/or additional drug use is permitted during the duration trial, if applicable. After the cessation of the trial, the patients’ usual clinical care can resume, if applicable. There is no anticipated harm or compensation for trial participation.

### Data collection

All collected data will be imputed and stored in an electronic case report form and stored securely on a password-protected encrypted computer.

#### MRI—primary objective

The MRI will be completed by a radiologist from the local radiology department. Imaging will be performed on a 3-Tesla high-resolution MRI (3 T-HR-MRI; Ingenia scanners, Philips Healthcare, Best, The Netherlands) with a dual surface coil (Sense Flex S; Philips Healthcare). After employing a survey to determine the carotid bifurcation position, the time of flight and T1- and proton density-weighted sequences will be acquired and centered on the identified carotid plaque perpendicular to the main carotid axis. Contrast-enhanced MR angiographic coronal images will be obtained before and during an injection of 30 mL of a gadolinium-based contrast agent (ProHance®, Bracco Imaging S.P.A., Colleretto Giacosa, Italy) followed by a 10-mL saline flush delivered at 2 mL/s. Finally, six T1-weighted slices will be repeated 5 min after the gadolinium injection, at the same level and with the same parameters as the T1-weighted pre-injection sequence.

Before semi-quantitative analysis, all images will be rated by experts (i.e., radiologist and vascular physician), in order to assess the image quality based on a 5-point scale dependent on the overall signal-to-noise ratio [34]. Images with a score of less than three will be excluded from the analysis. The images will be assessed by two experts independently, and both experts will be blinded identification factors. Disagreement between experts will be solved by consensus, if applicable. The degree of carotid artery stenosis will be assessed using MR angiography according to NASCET criteria. Fibrous cap rupture and large lipid cores (> 50% of the vessel wall thickness) will be rated as 0 (absent) or 1 (present). The presence of IPH and calcifications will be rated as 0 (absent), 1 (minor), 2 (moderate), or 3 (major). Thrombus will not be recorded. Juxtaluminal hemorrhages will be deemed as IPH, and the fibrous cap status will be recorded regardless of the presence or absence of IPH. Both experts will visually analyze signal changes in the vessel walls in the post-contrast images and will compare them to corresponding pre-contrast images and will complete contouring of the region of interest where uptake is found. Using in-house software for manual image registration, the mean signal intensity (SI) for each region of interest will be measured and normalized to the SI in the adjacent sternocleidomastoid muscle. The percentage of SI change will be automatically calculated as follows: (post-SI plaque × pre-SI muscle)/(pre-SI plaque × post-SI muscle). Precautions will be taken to exclude images with prominent flow artifacts. When pre- and post-contrast images cannot be co-registered, data will be excluded from the analysis. Gadolinium contrast enhancement will be characterized by its localization in the plaque (i.e., fibrous cap, shoulder region, or centrally located within the plaque).

#### Blood sampling

Blood will be sampled in one citrate tube, three ethylenediaminetetraacetic acid (EDTA) tubes, and two heparin tubes. Experiments will be performed within the first 4 h post-collection. One EDTA tube will be taken after an overnight fast and a sufficient period of time after any acute anti-inflammatory treatments, in order to determine lipidic content (i.e., total cholesterol, high-density lipoprotein cholesterol, low-density lipoprotein cholesterol, triglycerides), complete blood count (i.e., erythrocytes, leucocytes, neutrophils, lymphocytes, monocytes and platelet content, hematocrit), fasting glucose, and fibrinogen content.

Two 3-mL heparin tubes will be used to determine monocytic phenotype. Peripheral blood mononuclear cells will be isolated from whole blood using a Ficoll (Ficoll Paque PREMIUM, Cytiva, UK) gradient. Approximately 9.10^6^ peripheral blood mononuclear cells will be incubated for 1 h at 37 °C in a 12-well plate in a complete growing medium (95% RPMI 1640, 1% penicillin/streptomycin (10,000 U), 1% sodium pyruvate, 1% HEPES 1 M, 1% MEM vitamins 100X, 1% non-essential amino acids solution, 0.1% 2-mercaptothanol 50 nM), then washed with PBS to remove non-adhering lymphocytes. Roughly one-third of the monocytes will be saved for immediate labeling (see below) and the remaining monocytes will be incubated at 37 °C for 4 h both with and without lipopolysaccharide (LPS), after which point will be processed for labeling. The labeling procedure will include incubation with a Fc receptor blocking solution (FcR Blocking Reagent human, Miltenyi Biotech, Germany) for 30 min to saturate monocyte Fc receptors. FITC-conjugated anti-CD14, PE-conjugated anti-CD16 (both Miltenyi Biotech, Germany), and APC-conjugated anti-CD142 (eBioscince, MA, USA) antibodies will be added for 30 min at 4 °C. Controls included isotype-matched antibodies. The analysis will be performed using a BD FACSCanto II (Becton Dickinson, NJ, USA) flow cytometer.

One 4-mL EDTA tube will be used to analyze hematological and hemorheological parameters (Uyuklu et a. 2009, Connes et al. 2009 and Baskurt et al. 2009). Blood viscosity will be measured using a cone-plate viscometer (Wells-Brookfield, CPE-40 spindle, Canada), at 25 °C with different shear rates, from 11.25 to 225 s^−1^. Hematocrit levels will be determined using micro-centrifugation of a small glass capillary filled with 50–60 μL of whole blood. The hematocrit-to-blood viscosity, which corresponds to a hemorheological oxygenation index, will be calculated by dividing the hematocrit by blood viscosity at each shear rate. RBC deformability will be measured by ektacytometry (LORRCA®; Mechatronics Instruments BV, AN Zwaag, the Netherlands) at nine shear stresses ranging from 0.3 to 30 Pa at 37 °C. After normalization to 40% hematocrit, RBC aggregation and disaggregation thresholds (i.e., the strength of RBC aggregates) will be measured by syllectometry with the LORRCA® at 37 °C. All measures will be performed according to the international guidelines for hemorheological analysis [35].

One 3-mL citrated blood sample will be used for coagulation measurements within the first 4 h post-collection. A complete blood count will be done on total blood samples. Half of the blood will be used for ex vivo clot formation according to protocols from previous studies [36, 37] with and without tissue factor [38]. After a 2-h incubation at 37 °C, the formed clot will be weighed and a complete blood count will be done on the remaining serum in order to quantify RBC that were excluded from the clot. The remaining blood will be used to analyze clot kinetics with a rotative thromboelastometer ROTEM® (TEM International, Germany) in EXTEM mode [39]. Blood will be incubated for 1 h at 37 °C both with and without tissue factor in order to evaluate clot initiation, propagation, and lysis.

The last 4-mL EDTA tube will be immediately centrifugated for 10 min at 1500 rpm to collect plasma in 1-mL microtubes which will be frozen at − 80 °C for subsequent analysis. After all samples are collected, patients’ plasma will be used to determine the level of several oxidative stress markers (i.e., 8-hydroxy-2-deoxyguanosine, malondialdehyde, and advanced oxidation protein products), the activities of several antioxidant enzymes (i.e., superoxide dismutase, glutathione peroxidase, and catalase), and the nitric oxide metabolism-related products and cytokines (i.e., Interleukin (IL) 1β, TNFα, IL-4, IL-6, IL-10, and IL-12) will also be measured.

#### Physical activity fitness

The Alta HR FitBit device will be worn on the non-dominant wrist of patients for the 14 days following the clinical visits (#1 and #2). The connected device will be used to measure the daily step count achieved by each patient.

Considering skeletal muscle mass as an important factor in the maximal level of strength [40], thigh volume will be evaluated according to the international standards for anthropometric assessments [41]. Thus, lower limb strength will be measured using an isometric quadricep strength test (DFS II, Chatillon Force Measurement, AMETEK STC, USA). While sitting on a chair with a knee angle of 90° and a hip angle of 110°, the patients’ dominant leg will be immobilized and attached to a force gauge and the patient will be asked to proceed as if to complete a full extension of the leg. Patients will be asked to attempt to extend their leg one time for 3 s at 50 N, then at 75 N and 100 N as a warm-up. Between each test and after a 2-min interim recovery to avoid any skeletal muscle fatigue [42], the patient will be asked to extend three times as hard as possible for a 3-s duration. Patients will be encouraged to complete their best and highest performance [43, 44]. The highest measured value of maximal isometric quadricep strength will be expressed as a function of defatted dominant leg volume.

Walking endurance will be measured by the 6-min walking test (6MWT) which has been validated to reliably determine the maximum distance a patient is able to walk in 6 min [45]. Patients will be asked to walk as far as possible for 6 min between two cones placed 25 m apart in a straight corridor. After completing the walking task, patients will lay down for 15 min. At the end of the test, the total length walked (m) will be recorded. Borg’s perceived effort rate [46] (from 1 to 10), arterial oxygen saturation (SpO_2_, %), heart rate (bpm), and both systolic and diastolic blood pressures (mmHg) will be taken at three time points: [1] after a 10-min supine rest period, [2] immediately post-test in a supine position, and [3] 10 min after the end of the test. Oxygen uptake (VO_2_), expired carbon dioxide (VCO_2_), and ventilation will be recorded continuously using a face mask with a Metamax3b® analyzer (Cortex Biophysik, Leipzig, Germany), from 10 min before the 6MWT until 15 min after the 6MWT. The mean absolute (L/min) and relative (mL/min/kg) oxygen consumption and respiratory quotient will be measured at rest, at oxygen plateau during the 6MWT, and 10 min after the 6MWT.

#### Determination of lower extremity peripheral arterial disease (LE-PAD)

Identification of patients with LE-PAD is of interest as polyvascular atherosclerotic diseases disorders (i.e., carotid and peripheral artery disease) may induce more severe atherosclerotic effects [47] and mobility limitations []^-24^ as compared to patients with only carotid plaques. LE-PAD patients among the cohort will be identified using the ankle brachial index (ABI) calculated as the ratio between the anterior and posterior tibial arteries of each limb and the highest brachial systolic blood pressure. Arterial pressure of the anterior and posterior tibial arteries of both ankles will be measured using a Doppler probe (HUNTLEIGH Doppler Mini Dopplex D900, Sega Electronique, France). For each limb, the highest value of ABI will be considered, and the lowest of the two values will serve as the final ABI [48]. ABI measurements will be done first at rest and repeated after the 6MWT. A normal ABI value is within the range of 0.91 and 1.4.

#### Cognitive function

Since atherosclerotic patients may have impaired cognitive function [49, 50], cognitive capacities will be assessed using the Folstein mini-mental questionnaire [51] and, depending on patients performance, will necessitate the following questionnaires to be completed. A score will be derived by summing each point successfully answered, with scores ranging from 0 to 30. For those with a score greater than 27 [52], the following questionnaires will be completed.

#### Sedentary behavior

The daily amount of time spent being sedentary will be assessed by the Sedentary Behaviour Questionnaire [53]. Patients will be asked to estimate the time spent sitting or lying during nine activities (i.e., work, television, computer, meals, listening music, with friends/family, travels, leisure, and nap). Each answer will be summed to estimate the daily sitting time, expressed in min/day.

#### PA level and duration

Weekly PA levels and intensity will be assessed by the Global Physical Activity Questionnaire (GPAQ) characterizing walking trips or moderate to intense PA at work or during leisure time [54, 55] and expressed in [metabolic equivalent of task]-min/week.

#### Nutrition

The National Nutrition Health Plan (PNNS) questionnaire [56] will be used to characterize the quality and quantity of patients’ food intake [57], with scores ranging from 10 to 117.

#### Quality of life

Quality of life will be assessed using the EuroQol-5 Dimensions-5 Levels (EQ-5D-5L) questionnaire adapted for the French population in order to quantify mobility, autonomy, current activities, pain, anxiety, and depression [58] with scores ranging from 5 to 25 and a self-reported health evaluation ranging from 0 to 100%.

Tobacco consumption will be evaluated in a pack-year format as a function of daily consumption and total duration of active smoking.

#### Anthropometrics

Anthropometric characteristics including height (cm), weight (kg), skin fold (mm), and waist (cm) and hip (cm) circumference will be collected for each patient. Fat percentage will be calculated using the skin fold sum method equations [59] specifically for bicipital, tricipital, subscapular, and supra-iliac folds. Waist circumference will be measured around the abdomen at the midpoint between the last floating rib and the iliac crest. Hip circumference will be measured at the tip of the pubis. Body mass index will be calculated as the body weight in kilograms divided by the square of the height in meters (kg/m^2^).

#### Clinical data

Clinical data will be collected by the investigating surgeon, including date of birth, sex, resting heart rate, resting systolic and diastolic blood pressures, side, and percentage of stenosis. Personal medical history will be thoroughly gathered by the clinical research associate for any previous or current conditions such as acute coronary events, arterial retinal thrombosis, stroke, transient ischemic attack, myocardial infarction, peripheral arterial disease of the lower limbs, hypertension, type 2 diabetes, and/or dyslipidemia. The date of relevant events, duration, and associated treatments will also be reported. Additionally, patients will be asked for any known relevant familial medical history.

### Data management and monitoring

Throughout the trial, the sponsor of the project, the *Hospices Civils de Lyon* (contact: Director of Innovation and Research Department) will be responsible for the monitoring of patients.

### Collecting and data management

Patients will be identified using unique identification codes, including inclusion row, and the first letter of their first and last names. The investigator will be responsible for maintaining the anonymity of each patient in the study. Information will be collected for each patient at a defined timeline (Table 1) and reported in a standardized observation document filled out by the investigator or the co-investigator.

**Table 1:** Data collection schedule for the PACAPh study in both groups

	***Initial consultation***	***Visit #1***	***Follow-up phone calls***	***Visit #2***
	***D0***	***D7*** *± 7 D*	***Every 2 weeks***	***M6*** *± 15 D*
***Medical history***	*X*			
***Clinical data***	*X*			*X*
***Pregnancy tests*** *(only for females of child-bearing age)*	*X*			*X*
***Magnetic resonance imaging***	*X*			*X*
***Blood sampling***				
*Complete blood count and lipid content*		*X*		*X*
*Cytometry*		*X*		*X*
*Hemorheology*		*X*		*X*
*Coagulation analysis*		*X*		*X*
*Bioassays*		*X*		*X*
***Physical activity level***				
*Isometric quadricep strength*		*X*		*X*
*6MWT*		*X*		*X*
***Questionnaires***				
*Cognition (Folstein mini-mental)*		*X*		*X*
*Sedentary behavior*		*X*		*X*
*PA (GPAQ)*		*X*		*X*
*Nutrition (PNNS)*		*X*		*X*
*Quality of life (EQ-5D-5L)*		*X*		*X*
*Tobacco consumption*		*X*		*X*
***Anthropometrics***		*X*		*X*
***Compliance***			*X*	*X*
***Adverse event assessment***		*X*	*X*	*X*
***Activity tracker (PA group only)***				
*Steps/day*			*Continuously*	

The source documents include the original documents, data, and files, from which the data concerning the trial patients will be stored in an eCRF by the vascular surgery department clinical research associates. The investigator will be the sole person allowed to authorize access to the study source data during monitoring, audit, or inspection visits. Data from the FitBit®, Metamax®, flow cytometry, ROTEM®, and LORRCA® will be extracted from the local password-protected computers and stored on a LIBM password-protected computer that can be accessed only by registered investigators (MM, LDK, JL, VP, AM). All row data will be checked monthly by the investigator to ensure that all protocols and ethical guidelines for data collection and analyses are followed. An interim analysis will be done by the statistician (VP) after half of the patients finished the protocol (*n* = 40) and again after every 10 additional patients. If the primary objective is reached (i.e., decrease in IPH scores in the PA group), the study will be terminated by the decision of the principal investigator (AM). Study data will be entered through the eCRF hosted on the ENNOV CLINICAL software, which allows for real-time data quality control. In order to meet the regulatory requirements, the software complies with the recommendations concerning computerized systems for management of clinical trials and electronic signature and standards. Connection to such server is made through the use of a unique password and identifier specific to each user, which will only give access to patient data. An audit function can be integrated to the software, allowing traceability of the date collected as well as any further modifications completed. The encrypted data will then be transmitted to the department responsible for data management via a secure internet connection.

The individual data necessary for the analysis will be as follows:

- Entered into eCRF as obtained, whether they are clinical or para-clinical data
- Anonymized by the investigator
- All filled-in; missing data must be justified

Firstly, the data will be checked and validated by the clinical research associates or the investigator from the source document. The data manager will use computerized consistency tests in order to flag the presence of non-standard, missing, aberrant, or inconsistent data throughout the data entering process. Each inconsistency identified in the eCRF will be subject to a request for a modification that must be justified. Once the required data are entered, checked, and validated, the study database will be locked. The maintenance and security of the database will be the responsibility of the Innovation and Research Department of the study sponsor.

The computer database and any modifications will be saved and available by the Innovation and Research Department of the *Hospices Civils de Lyon*. The locking and exportation of the database will be done in accordance with the usual procedure of the Innovation and Research Department.

### Monitoring and quality control

An independent data and safety monitoring board will be formed for any potential pathophysiological and/or PA issues and will be operated by the study promoter. Members will not have any financial or scientific conflicts of interest with the PACAPh trial. The monitoring board will be composed of clinical research associates with experience in clinical trial monitoring. The objective of the monitoring board will be to ensure the safety of trial patients as well as maintain the scientific integrity of the trial by monitoring the data. The monitoring board will aim to meet approximately every 6 months during the trial period. There are no pre-defined statistical guidelines for the premature termination of the study. As the risk to develop serious adverse events (AE) linked to the intervention are low, no steering committee was planned.

### Adverse event reporting and harms

Any AE or serious AE (i.e., any incidents leading to hospitalization) that occur from the signing of the consent to the exiting of the study will be reported by an investigator to the Innovation and Research Department by filling out an incident report form. The Innovation and Research department will (1) assess the causal link between the protocol and the AE and/or serious AE, (2) assess whether the AE is expected or unexpected (i.e., defined in the protocol), and (3) declare all serious and unexpected AE within the regulatory deadlines to the Health Authorities and the Ethics Committees concerned and inform the investigators.

Study-related and serious AE including all ischemic events, falls leading to injuries such as sprains or broken bones whether or not requiring hospitalization or leading to death, and AE not related to the study such as household accidents (e.g., falls, cuts) will be recorded during the trial. All types of AE occurring during the study will be followed-up until resolution, as necessary. The study will be paused immediately if reports of a serious AE are filed and will not resume until after inquiry from the promoter. If it is determined that the study is not the cause of the serious AE, the study will resume. If the study is determined to be the cause of the serious AE, the study will be terminated.

### Statistical analysis

#### Sample size

Based on the results of Mury et al. 2019 [18], the intended samples size was calculated using G*power for between-factor repeated measures. An a priori power calculation based on a medium effect size (*f* = 25, *α* = 5%, 1−*β* = 0.85) resulted in the requirement of 74 patients. Considering an 8% attrition rate, we will aim to recruit 80 patients.

#### Statistical methods for primary and secondary outcomes

Analyses will be performed on the intent-to-treat population. Patient characteristics will be described at visits #1 and #2 using the mean and standard deviation, and frequency and percentage for continuous and discrete data, respectively. For the primary outcome, the evolution of IPH as determined through MRI between baseline (visit #1) and the end of the study (visit #2) in both groups will be evaluated using a two-way analysis of variance (ANOVA). For the secondary outcomes, time and group effects will be analyzed. Two-way ANOVA with Bonferroni post hoc corrections will be used to explore the potential effects of PA on quantitative variables. A *χ*^2^ test will be used to analyze the effects on qualitative data. In addition, biological, psychological, and clinical outcomes will be analyzed using a multiple linear regression considering potential confounders such as age, biological sex, treatment (PA vs. control), and comorbidities. All statistical analyses will be performed using the Rstudio software (1.8.2, Boston, MA, USA). The significance level will be set at *p* < 0.05.

An adherence-adjusted analysis will be performed on the outcomes if there is more than 4% non-adherence to intervention (i.e., non-achievement of final PA steps/day goals) or differential adherence across intervention (i.e., increase, stabilization or a decrease in steps/day count). An analysis of missing data at visit #2 will be performed in order to determine factors associated with the missing data profile. Multiple imputation methods and sensitivity analysis (i.e., propensity score) will be performed for missing data to elucidate the potential influence of missing responses. All analysis, including methods used to handle missing data, will be fully described in the statistical analysis plan.

### Ethics and reglementary aspects

The research will be conducted in accordance with the French regulations in the application, in particular, the provisions relating to research involving humans: Law No. 2012 − 300 of March 5, 2012, relating to research involving humans and its implementing decrees (Decree No. 2016 − 1537 of November 16, 2016, relating to research involving humans, Decree No. 2017 − 884 of May 9, 2017, amending certain regulatory provisions relating to research involving humans), the laws of Bioethics (if applicable), Law No. 78 – 17 of January 6, 1978, as amended, relating to Computing, Files and Freedoms, the Declaration of Helsinki, and Good Clinical Practices.

The information intended for trial patients will include all elements defined in Law No. 2012 – 300 of March 5, 2012, relating to research involving the human person (*Jardé* Law) and will be written in layman’s terms and in a language understandable to the patients. The sponsor of the study, *Hospices Civils de Lyon*, has taken out a civil liability insurance contract for the entire duration of the study with the *Société Hospitalière d’Assurance Mutuelle* guaranteeing its own civil liability as well as that of any party involved in the conduct of the trial, regardless of the nature of the links between the patient and the sponsor.

The data recorded during this research are subjected to computer processing under the responsibility of the *Hospices Civils de Lyon*, the sponsor, in accordance with the law n ° 78 − 17 of January 6, 1978, as amended relating to the information technologies, files, and freedoms.

## Discussion

Within the last decade, it has been suggested that CEA surgery is not appropriate or necessary for all asymptomatic stroke patients [4]. According to a recent meta-analysis [60], at least half of males aged 65 years old and older have carotid plaques. Thus, there is a new category of patients that present with detectable carotid stenosis, yet will not undergo CEA surgery based on the low/mild severity of their plaque vulnerability. At this point, secondary preventative measures should aim to maintain plaque stability and limit the degradation of the plaque at risk of rupture. It is suggested that PA should be encouraged for these patients to maintain carotid plaque stability, on the basis of lower histologic IPH in patients with higher levels of PA [18]. However, these recommendations are mainly based on the results of cross-sectional studies. Therefore, this highlights the need for a longitudinal intervention study to provide the strongest and most accurate level of proof to validate the beneficial effects of PA in this population. To our knowledge, this study will be the first longitudinal trial that aims to assess the effects of a PA intervention using walking practices on plaque stability and MRI to assess such changes in carotid atherosclerotic patients.

We hypothesize that a home-based PA intervention will reduce IPH in carotid atherosclerotic patients who have not undergone CEA surgery. If our hypothesis is confirmed, benefits for patients may include improvements in stroke risk factors through decreasing IPH [18] as well as circulating factors associated with negative prognoses [19, 20, 61]. Moreover, according to previous cross-sectional studies [19, 20], moderate PA may be more beneficial on IPH and monocytic phenotypes than intense PA for these patients, suggesting walking as an important therapy tool for this population. Similarly, moderate-intensity PA may be more efficient in reducing the pro-thrombotic state in coronary patients as compared to high-intensity PA [14]. Patients may also have improved quality of life and autonomy by increasing their walking ability and distances around their respective communities. In addition, we hypothesize that LE-PAD cohort patients will not respond in a similar fashion as compared to non-LE-PAD cohort patients regarding clinical parameters and carotid plaque features. Given the high rate of instability in carotid plaques (39.5%) observed in lower extremity peripheral arterial disease patients [62], the use of a systematic evaluation of vascular lower limbs state by ABI and personalized PA programs in these patients would be useful to individualize their therapeutic care.

To our knowledge, MRI has not yet been used in a longitudinal study aimed at assessing the evolution of carotid IPH and other instability criteria (i.e., calcifications and lipidic core volume). In a recent review [63], we proposed that MRI is likely the most effective way to diagnose in vivo carotid IPH. The results of this study should therefore provide information on the relevancy and efficiency of MRI for the monitoring of carotid plaque IPH in response to PA interventions.

Our PA program is based on home-based cardiovascular rehabilitation. Implemented at a larger scale, home-based cardiovascular rehabilitation could thus blunt the disparities in the care, such as work commitments or distance from the hospital [64], and service accessibility especially for those in rural communities [65]. Moreover, as elderly patients are more subject to social isolation, especially during the current COVID-19 pandemic situation, frequent phone calls may increase their social interaction and thus their overall morale and feelings of well-being. In addition, we will use connected activity trackers to follow and create PA goals for patients. Connected devices can be programmed to provide reminders of the daily goals and can be set to specifically fit within patient capacities, needs, and goals. However, although home-based PA programs may be an efficient way to maintain activity for cardiovascular patients [66], without appropriate follow-up assessments, adherence may suffer from the lack of supervision. Our intervention is well designed in order to avoid these limitations. Indeed, our intervention combines connected devices and bi-monthly phone calls, which will include the setting of new daily steps goals and motivational speeches. Regular re-evaluations of daily step count goals may be an easy tool to ensure PA adherence in these patients. Further, empowering patients through the use of verbal motivation and encouragement is an extremely effective tool that can assist individuals in regaining control and ownership of their lives. It may improve self-confidence, motivation, proactivity, and capacity to act, allowing patients to become self-sufficient and self-reliant in the management of their illness, treatments, and more precisely, in the completion of their PA program [67]. Moreover, if the results of PACAPh study show beneficial effects on the outlined health parameters, the implementation of such kind of an intervention in the daily care of these patient at a large scale would be highly feasible and possess a very advantageous cost-effective ratio.

Finally, this research may allow for PA recommendations to be added to the medical guidelines and advice given to asymptomatic patients with marked carotid stenosis who do not undergo carotid surgery. Atherosclerotic patients are an elderly population more subject to social and geographical isolation. Therefore, compared with an intervention completed within a rehabilitation center, our home-based PA intervention will be more acceptable for these patients and their long-term health.

## Dissemination plan

The results derived from the primary and secondary outcome analyses of this project will be reported in peer-reviewed journal articles. The results will also be presented at leading national and international conferences and in the PhD thesis of MM. The results will be communicated to health professionals and participants through meetings and email/letter, respectively.

## Trial status

The protocol version currently used is the 4th initially accepted on September 09, 2021, and modified on February 26, 2021. Recruitment began in December 2019 and will be completed in February 2022.

## Data Availability

MM, ER, LDK, AM, and VP will have access to the final trial dataset. Raw data are registered in an electronical case report form (eCRF; ENNOV Clinical, CSOnline 7.5.720.1). The datasets used and/or analyzed during the current study are available from the corresponding author on reasonable request.

## Abbreviations

ABI: Ankle brachial index
AE: Adverse event
CEA: Endarterectomy surgery
EDTA: Ethylenediaminetetraacetic acid
eCRF: Electronic case report form
IL: Interleukin
IPH: Intraplaque hemorrhage
LE-PAD: Lower extremity peripheral arterial disease
MRI: Magnetic resonance imaging
PA: Physical activity
RBC: Red blood cell
6MWT: Six-minute walking test
