# Correction to: Effectiveness of an individualized home-based physical activity program in surgery-free non-endarterectomized asymptomatic stroke patients: a study protocol for the PACAPh interventional randomized trial

**DOI:** 10.1186/s13063-022-06135-w

**Published:** 2022-03-22

**Authors:** Mathilde Mura, Emeraude Rivoire, Leila Dehina-Khenniche, Michèle Weiss-Gayet, Bénédicte Chazaud, Camille Faes, Philippe Connes, Anne Long, Chantal L. Rytz, Pauline Mury, Lidia Delrieu, Etienne Gouraud, Marine Bordet, Nellie Della Schiava, Patrick Lermusiaux, Matthieu Arsicot, Antoine Millon, Vincent Pialoux

**Affiliations:** 1 Atherosclerosis, Thrombosis and Physical Activity, LIBM EA7424, Université Lyon 1, University of Lyon, Lyon, France; 2 Vascular Medicine Department, Hopital Edouard Herriot, Hospices Civils de Lyon, Lyon, France; 3 Vascular and Endovascular Surgery Department, Hopital Louis Pradel, Hospices Civils de Lyon, Lyon, France; 4 Stem Cell Environment and Skeletal Muscle Homeostasis, Institut NeuroMyoGene, CNRS UMR 5310, INSERM U1217, Université Claude Bernard Lyon 1, University of Lyon, Lyon, France; 5 Vascular Biology and Red Blood Cell, LIBM EA7424, Université Lyon 1, University of Lyon, Lyon, France; 6 Libin Cardiovascular Institute, Cumming School of Medicine, University of Calgary, 3230 Hospital Drive NW, Calgary, Alberta Canada; 7 Center for Research, Montreal Heart Institute, Montreal, Quebec, Canada; 8 Residual Tumor & Response to Treatment Laboratory, RT2Lab, Translational Research Department, INSERM, U932 Immunity and Cancer, Institut Curie, Paris University, Paris, France; 9 Electrical Engineering and Ferroelectrical Laboratory, INSA Lyon, Lyon, France

**Correction to: Trials 23, 145 (2022)** **https://doi.org/10.1186/s13063-022-06061-x**

Following the publication of the original article [1], we were notified that one of the author names has been incorrectly tagged.

Original tagging:

- First Name: Nellie Della
- Last name: Schiava

Corrected tagging:

- First Name: Nellie
- Last name: Della Schiava

The original article has been corrected.
